# People with long-term conditions are more adherent to protective behaviours against infectious disease

**DOI:** 10.1016/j.puhip.2024.100538

**Published:** 2024-08-22

**Authors:** Gill Hubbard, Diane Dixon, Marie Johnston, Chantal den Daas

**Affiliations:** a School of Health Sciences, 11 Airlie Place, University of Dundee, Scotland, United Kingdom; b Health Psychology Group, University of Aberdeen Institute of Applied Health Sciences, Aberdeen, Scotland, United Kingdom; c School of Applied Science, Edinburgh Napier University, Edinburgh, Scotland, United Kingdom

**Keywords:** COVID-19, Infectious disease, Chronic disease, long-term conditions, health behaviour

## Abstract

**Objectives:**

To investigate the relationship between long-term condition (LTC) status and adherence to protective behaviours against infectious disease (face covering, physical distancing, hand hygiene).

**Study design:**

Representative cross-sectional observational survey in summer 2020 in Scotland.

**Methods:**

Independent variable is LTC status (LTC, disability, no LTC); dependent variable is adherence to protective behaviours (face covering, hand hygiene, social distancing); moderator variables are age, gender and area deprivation; mediator variables are perceived threat and psychological distress. P values of p < 0.05 were taken as statistically significant.

**Results:**

3972 participants of whom 2696 (67.9 %) indicated not having a LTC. People with no LTC had lowest adherence to protective behaviours, perceived threat and psychological distress. Age did not moderate the relationship between LTC status and adherence; females were more adherent than males and this gender difference was greater in people with disability compared to people with no LTC; adherence was greater for people with a LTC in the more deprived areas compared to the least deprived areas whereas adherence in those with no LTC was not related to area deprivation; threat appraisal partially mediated the relationship between having a LTC or disability and adherence; psychological distress did not mediate the relationship between LTC status and adherence.

**Conclusions:**

This study addresses a gap in evidence about protective behaviours of people with LTCs. Perceptions of threat may be useful intervention targets against winter flu and during future pandemics in order to protect people with LTCs who are one of the most vulnerable groups of the population.

## What this study adds

• People with a LTC are more likely to adhere to behaviours that will protect them from infectious diseases, such as COVID-19 and influenza compared to people with a LTC
• People with a LTC are more likely to perceive that they were at greater risk from infectious diseases such as COVID-19 and influenza compared to people with a LTC

## Implications for policy and practice

• People with a LTC are likely to be receptive to public health messaging about protective behaviours from infectious diseases.
• The moderating effects of sociodemographic factors (gender and area deprivation) suggest interventions that aim to increase adherence in people with a LTC might want to target specific groups in the population that are disproportionally affected by infectious diseases, such as people living in deprived compared to affluent areas.
• In addition, our study suggests that threat appraisal is likely to be an important intervention component because it partially mediates the relationship between LTC status and adherence. Tailored persuasive communication for instance, may include threat messages that do not provoke psychological distress in order to influence adherence in people with LTC(s).

## 1 Introduction

Long-term conditions (LTC) are a public health priority. A quarter of the population in the United Kingdom and over one-third of people in the European Union have a LTC [[1], [2], [3], [4]]. The prevalence of LTCs is higher in people who are aged 65 and over and the onset of multimorbidity (one or more LTC) occurs 10–15 years earlier in people living in the most deprived areas compared with the most affluent areas [5]. A LTC is associated with increased risk of severe influenza disease [6] and people with a LTC had a higher risk of developing COVID-19 [7], increased mortality [8,9], risk of hospitalisation [10,11], and negative clinical outcomes [12,13]. Understanding adherence to protective behaviours such as physical distancing, face covering, and hand hygiene in people with a LTC and understanding factors that influence these protective behaviours in this group of the population will contribute to efforts to protect this group against infection and in potential future pandemics.

Psychological theories have been used to explain why certain groups of the population are motivated towards and more adherent to protective behaviours than others [14]. A review of the application of behaviour change theories within an infectious disease and emergency response context identified the three most commonly cited theories, namely, the Health Belief Model (HBM), Protection Motivation Theory (PMT), and the Theory of Planned Behaviour (TPB) [15]. Both HBM and PMT propose *threat appraisal* as a key cognitive motivational determinant for adopting protective behaviours [16,17]. Whether these motivational beliefs are stronger in people with a LTC compared to the general population and influence adherence to protective behaviours merits investigation because it can inform for instance, winter flu interventions targeting these groups.

Besides different experiences of threat due to having a LTC, emotional responses to the threat, such as psychological distress may also explain some of the variance in protective behaviours in people with a LTC. A cross-sectional study of people with hypertension and diabetes found that anxiety about contracting COVID-19 and about death due to COVID-19 were associated with increased handwashing before eating, after using the restroom, after returning from outdoors, for at least 30 seconds (s) and with soap or hand sanitizer [18]. Another study found that people with anxiety-related or mood disorders were more likely to voluntarily self-isolate than those without a mental health disorder [19]. Hence, adherence to protective behaviours may be influenced by increased levels of anxiety with these behaviours serving as active coping responses to reduce either the experience of anxiety and/or the threat of the COVID-19 to physical health.

### 1.1 Aims and hypothesises

**Fig. 1:**
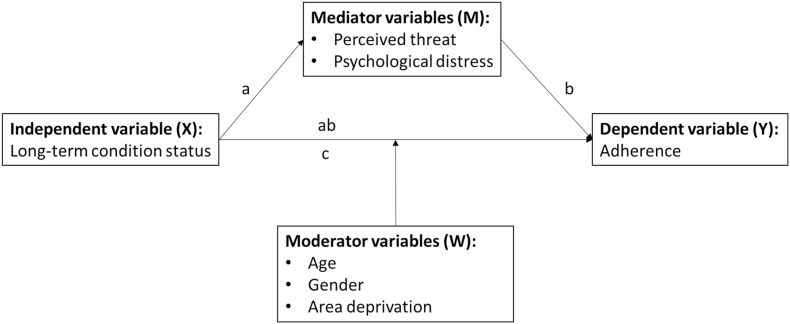
Theoretical representation of the proposed simple mediation and moderation models including the pathways for testing mediation (a,b,c,ab).

In this study, we aimed to find out if people with a LTC were more adherent to protective behaviours during the COVID-19 pandemic than those without a LTC. The study addresses an important gap in public health research. The hypothesised pathways linking LTC with psychological and sociodemographic factors and behaviour are illustrated in Fig. 1.

## 2 Methods

### 2.1 Design and setting

A serial weekly nationally representative cross-sectional observational study of approximately 500 randomly selected adults in Scotland over 8 weeks was conducted in summer 2020. No respondent took part in several surveys. The survey was administered by telephone by Ipsos MORI (a commercial polling company).

### 2.2 Participants

Adult men and women aged 16 or older, able to speak English, and currently living in Scotland were eligible. No other exclusion criteria were applied. Ipsos MORI sampled participants using random digit dialling to landlines and mobile phones. Quotas on gender, age, working status, and geographical regions were applied to achieve a representative sample.

### 2.3 Variables and measures

#### 2.3.1 Independent variable

LTC status was defined and measured using items from the UK Census [20]: “Do you have a physical or mental health condition or illness lasting or expected to last 12 months or more?” Participants were given the following response options: yes, no, don't know, prefer not to say. Participants who answered yes were asked a follow-up question: “Does your condition or illness reduce your ability to carry-out day-to-day activities?” with the following four response options: yes a lot, yes a little, no, don't know, prefer not to say. We used responses to these questions to categorise participants into one of three mutually exclusive groups based on LTC status: (i) without a LTC (no LTC) (ii) a LTC(s) that did not affect their day-to-day activities (LTC), and (iii) a LTC(s) that affected their day-to-day activities a lot or a little, i.e. a life limiting long-term condition (disability).

#### 2.3.2 Dependent variable

##### 2.3.2.1 Adherence to protective behaviours

One overall adherence score (adherence) was calculated using a weighted sum-score using the Transmission-Reducing behaviour Adherence Measure (TRAM) based on evidence showing that transmission-reducing behaviours can be treated as one factor [21]. The individual adherence behaviours contributing to the TRAM were as follows: Physical distancing (assessed with one behaviour: staying 2m (6 feet) away from other people); wearing a face covering (assessed in relation to two environments: wearing a face covering when in a shop or when travelling on public transport); handwashing (assessed with four behaviours: washing hands as soon as you get home; washing hands using soap and water; washing hands for at least 20 s and washing hands before eating and drinking). Participants indicated the extent to which they had adhered to each behaviour over the previous week using a 5-point response scale (always [5], most times [4], sometimes [3], rarely [2], and never [1]).

#### 2.3.3 Moderator variables

*Age* was assessed continuously in years. *Gender* was assessed using Office for National Statistics categories, and for analyses we re-coded to binary categories (0 = female, 1 = male). *Socioeconomic area deprivation* was assessed using the Scottish Index of Multiple Deprivation (SIMD) [22]. All 6976 data zones were grouped into 10 bands (deciles); decile 1 contains the 10 % most deprived data zones in Scotland and decile 10 the 10 % least deprived data zones in Scotland. The deciles were used as a continuous variable in the analyses. *Employment status* was assessed using 11 categories and for analyses we re-coded to four categories (0 = full-time working, 1 = part-time working, 2 = not working, 3 = students).

#### 2.3.4 Mediator variables

*Perceived threat* was measured by two items: “If you were ill with COVID-19 it would be serious for you; ” and “It is likely that you will get COVID-19”. There were four responses ranging from ‘strongly agree’ to ‘strongly disagree’, and with the option of responding ‘don't know’ or ‘prefer not to say.’ In line with PMT, we multiplied the measures of perceived severity (scale 1–4) and vulnerability (scale 1–4), to produce an overall perceived threat score (range 1–16) [23]. The composite score indicates the combined threat of these two distinct dimensions of threat perception, which is a key motivational determinant of behaviour.

*Psychological distress* was measured using the 4-item Patient Health Questionnaire (PHQ-4), which is a brief screening scale for anxiety and depression [24]. Participants were asked: “Over the last 2 weeks, how often have you been bothered by the following problems? Tell me which answer option best applies: (1) Feeling nervous, anxious, or on edge (2) Not being able to stop or control worrying (3) Feeling down, depressed, or hopeless (4) Little interest or pleasure in doing things”. For each item, participants were given the following response options: Not at all, several days, more than half of the days and nearly every day. The total score ranges from 0 to 12 with categories of psychological distress being none 0–2, mild 3–5, moderate 6–8 and severe 9–12. The anxiety subscale is the sum of items 1 and 2 and the depression subscale is the sum of items 3 and 4. On each subscale, a score of 3 or greater is considered positive for screening for anxiety and depression purposes [24]. We made this into a categorical variable to indicate whether participants had either anxiety or depression or both (psychological distress), or neither (no psychological distress).

### 2.4 Statistical methods

For all variables, answers ‘I don't know’ and ‘I prefer not to say’ were treated as missing values and excluded from the analyses. Participants with missing data are not included in the analyses for example, we treat participants who did not report their SIMD as missing and therefore did not include them in the analyses of the moderation between SIMD and LTC status. P values of p < 0.05 were taken as statistically significant. Univariate ANOVA was used to analyse associations between LTC status and adherence. Moderation model analyses with Hayes' PROCESS macro (v 3.5, model 1) [25] was used to assess if socio-demographic factors moderated the relationship between LTC status and adherence. Models were tested in two steps. In the first step, LTC status was entered with one of the moderator variables (age, gender or Scottish Index of Multiple Deprivation). In the second step of the regression analyses, the interaction term between the moderator and LTC status was entered. For the analyses, a 95 % bias-corrected percentile bootstrapped confidence interval (CI) method was used, and 5000 bootstrap re-samples were produced for moderation examination. Additionally, we employed conventional methods for plotting simple slopes to understand moderation effects, at one standard deviation below and above the mean [26]. Mediation analyses with Hayes' PROCESS macro (v 3.5, model 4) [25] was used to assess if LTC status affected adherence *through* perceived threat or psychological distress. We used bootstrapping (10,000 samples) to analyse the extent to which the effect of LTC status affected perceived threat or psychological distress, and through these mediators, affected adherence. In this procedure, total effects, direct effects and indirect effects are estimated by means of ordinary least squares (OLS) regression analyses. The effect of the independent variable is displayed in the total effect, when controlling for the mediator variable it is indicated in the direct effect. The indirect effect comprises the path over the mediator variable. All statistics were performed using IBM SPSS Statistics.

## 3 Results

### 3.1 Participants

In total 3972 participants took part of whom 2696 (67.9 %) indicated not having a LTC, 734 (18.5 %) participants indicated they did have a LTC which did not affect their ability to carry out day-to-day activities, 512 (12.9 %) participants indicated to have a LTC that reduced their ability to carry out day-to-day activities a lot or a little (i.e. disability), and 30 (.8 %) participants indicated they did not know or preferred not to answer these questions (Table 1). People with LTC or disability were more likely to be older (>55 years) compared to people with no LTC. People with a LTC were more likely to be female and in lower SIMD deciles compared to people with no LTC or disability.

**Table 1:** Sociodemographic characteristics of participants with no long-term condition (no LTC), a long-term condition (LTC), and a limiting long-term condition (disability).

		No LTC		LTC		Disability	
		N	%	N	%	N	%
Total		2696	100	734	100	512	100
Age	Mean (SD)	49.9 (17.6)		54.5 (17.8)		56.5 (17.5)	
Age	16–24	292	10.8	60	8.2	28	5.5
	25–34	389	14.4	75	10.2	47	9.2
	35–44	367	13.6	80	10.9	52	10.2
	45–54	493	18.3	119	16.2	74	14.5
	55–64	530	19.7	158	21.5	121	23.6
	65+	625	23.2	242	33.0	190	37.1
Gender	Male	1108	41.1	248	33.8	220	43.0
	Female	1583	58.7	484	65.9	290	56.6
	Missing	5	.2	2	.3	2	.4
SIMD	Mean (SD)	6.4 (2.7)		5.4 (2.8)		6.3 (2.8)	
SIMD	1	111	4.1	65	8.9	22	4.3
	2	151	5.6	62	8.4	34	6.6
	3	164	6.1	66	9.0	45	8.8
	4	188	7.0	70	9.5	30	5.9
	5	223	8.3	55	7.5	38	7.4
	6	267	9.9	73	9.9	57	11.1
	7	285	10.6	84	11.4	57	11.1
	8	321	11.9	77	10.5	46	9.0
	9	313	11.6	59	8.0	65	12.7
	10	351	13.0	46	6.3	67	13.1
	Missing	322	11.9	77	10.5	51	10.0
Employment	Full-time	1026	38.1	122	16.7	133	26
	Part-time	298	11.1	60	8.2	59	11.5
	Unemployed	1089	40.4	496	67.8	277	54.2
	Student	280	10.4	54	7.4	42	8.2
	Missing	3	.1	2	.3	1	.2

### 3.2 LTC status and behaviour adherence

A univariate ANOVA showed that LTC status was associated with adherence to protective behaviours, *F* (2, 3939) = 102.107, *p* < 0.001, _*p*_*η*^*2*^ = .049. People with no LTC had lowest adherence (M = 14.982, SD = 1.852), followed by people with disability (M = 15.375, SD = 1.896), followed by people with a LTC (M = 16.088, SD = 1.934). All three groups significantly differed from each other (all p's < .001).

### 3.3 LTC status and perceived threat

A univariate ANOVA showed that LTC status was associated with perceived threat, *F* (2, 2959) = 32.509, *p* < 0.001, _*p*_*η*^*2*^ = .022. People with no LTC had lowest perceived threat (M = 6.269, SD = 3.005, both p's < .001), compared to people with a LTC or disability. There was no statistically significant difference between people with a LTC and disability (M = 7.021, SD = 3.121, and M = 7.388, SD = 3.382 respectively, p = 0.179).

### 3.4 LTC status, sociodemographic factors and behaviour adherence

Table 2 summarises the statistically significant moderator analyses for adherence.

**Table 2:** Moderator analyses of the relationship between LTC status and adherence.

Moderator	F	R^2^	ΔR^2^
Age*LTC status	(2, 3902) 62.599	.0743	.0.0011
Gender*LTC status	(2, 3927) 2.2776	.0694	.0011
SIMD*LTC status	(2, 34,486) 8.7746***	.0547	.0048

a Age = 1SD below mean age, SIMD = 1SD below deprivation mean (more deprived).

b Age = 1SD above the mean age; 1SD above deprivation mean (less deprived).

*p ≤ 0.05; **p ≤ 0.01; *** ≤ 0.001***p ≤ 0.001

Overall, age did not moderate the relationship between LTC status and adherence. There was a main effect of age; as age increased adherence also increased for people with a LTC, disability and no LTC (B = .0159, SE = .0020).

**Fig. 2:**
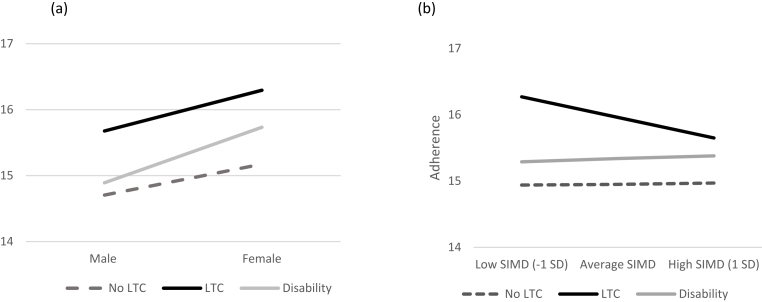
Graphical representation of the moderations between (a) gender and (b) socioeconomic area of deprivation (SIMD) and long-term condition status on adherence.

Overall, gender did not moderate the relationship between LTC status and adherence. There was a main effect of gender; males were less adherent than females (B = −.4689, SE = .0726). When assessing simple slopes, we did see an interaction between LTC status and gender (Fig. 2). When comparing people with disability and people with no LTC we found for both groups that females were more adherent than males and this gender difference was greater in people with disability compared to people with no LTC (B = −.3730, SE = .1810). No gender moderation effects were observed when comparing people with a LTC and people with no LTC.

Overall, area deprivation moderated the relationship between LTC status and adherence (B = −.1198, SE = .0297). When comparing people with a LTC and people with no LTC we found that adherence in people with a LTC was related to area deprivation whereas adherence in those with no LTC was not related to area deprivation; for people with a LTC adherence was greater in the more deprived areas compared to the least deprived areas (Fig. 2). No area deprivation moderation effects were observed when comparing people with disability and people with no LTC.

Overall, employment status did not moderate the relationship between LTC status and adherence. There was a main effect of employment status on adherence; people who were unemployed were more adherent than people who were full-time employed (B = .4842, SE = .0808). When assessing simple slopes, we did not see any interactions between LTC status and employment status.

### 3.5 LCT status, psychological factors and behaviour adherence

LTC status was related to threat appraisal, F (2,2959) = 32.509, p < 0.001. Compared to people with no LTC, people with a LTC or disability had greater threat appraisal (Path a: unstandardised B = 1.119, SE = .150, 95 % Confidence Interval CI = [.72–1.41], and unstandardised B = .752, SE = .172, 95 % CI = [.41–1.09] respectively). Threat appraisal was related to adherence (Path b: unstandardised B = .10, SE = .01, 95 % CI = [.08, .12]). The indirect effects (denoted as Path ab in Fig. 1) of LTC status on adherence via threat appraisal were significant (Path ab: unstandardised B LTC = .11, *SE* = .02, 95 % CI = [.07, .15] and unstandardised B LLTC = .07, *SE* = .02, 95 % CI = [.04, .11]). The direct effects were also significant (Path c: unstandardised B LTC = .88, *SE* = .09, 95 % CI = [.71, 1.06] and unstandardised B LLTC = .24, *SE* = .10, 95 % CI = [.04, .44]). Taken together, threat appraisal partially mediated the relationship between having a LTC or disability and adherence.

LTC status was associated with psychological distress, F (2,3939) = 310.490, p < 0.001. Compared to people with no LTC group, people with LTC or disability had greater psychological distress (Path a: unstandardised B = 2.601, SE = .104, 95 % CI = [2.40–2.81], and unstandardised B = .5174, SE = .121, 95 % CI = [.28-.75] respectively). The path (Path b in Fig. 1) between psychological distress and adherence was not significant (p = 0.131). The indirect effects (denoted as *ab* in Fig. 1) of LTC status on adherence via psychological distress were not significant either. Taken together, psychological distress did not mediate the relationship between LTC status and adherence.

## 4 Discussion

We found that people with a LTC or disability were more adherent than people with no LTC and perceived threat was higher in people with a LTC or disability compared to people with no LTC. These differences were greater in females and people in more deprived areas. Neither age nor employment status moderated the relationship between LTC status and adherence. Perceived threat partially mediated the relationship between LTC status and adherence. Psychological distress had no mediating effect.

Given that people with LTCs had a higher risk of developing COVID-19 [7] and a greater chance of mortality [8,9] it is perhaps not surprising that we found that people with a LTC were more adherent, had higher perceived threat, and psychological distress compared to people with no LTC. Studies conducted during the COVID-19 pandemic show that females were more adherent than males [27]. We therefore expected that the positive association between LTC status and adherence would be greater in females compared to males and this is what we found in people with disability compared to people with no LTC. Our study does not examine why gender had a moderating effect but other literature suggests that our findings may reflect gender differences in the lived experience of illness. A qualitative study of the experience of multiple chronic conditions in later life found that women were concerned about how their illnesses might affect their significant others such as family members, thereby responding to feminine norms of selflessness, sensitivity to others and nurturance whereas men's stories reflected masculine norms of control, invulnerability, physical prowess, self-reliance and toughness [28]. There are also gender differences in cognitions which may aid understanding why females were more adherent during the COVID-19 pandemic than males; studies have shown for instance, that perceived threat was higher in females and they were more stressed, worried and afraid than males and these cognitions were associated with greater adherence [29,30].

We expected that the positive association between LTC status and adherence would be greater in the most deprived areas compared to the least deprived areas. As expected, this was the case for people with a LTC although unexpectedly, not for people with disability. Our hypothesis was premised on studies which found that the most deprived areas had the highest number of COVID-19 cases [31] and the highest rates of death attributable to COVID-19 [32] and incidence of multimorbidity (one or more LTC) is significantly higher in the most deprived areas [33]. Thus, there was a greater real threat from COVID-19 in the most deprived areas, which we expected to translate into greater adherence in people with a LTC or disability in these areas. There is little data on the psychological mechanisms that may explain why area deprivation had a moderating effect on the relationship between LTC status and adherence and few studies investigating the interaction effects of area deprivation on protective behaviours during the COVID-19 pandemic. One study found that keeping 2 metre physical distance when outside the home was higher in the more affluent areas than the most deprived areas [34] whereas our study suggests that at least for people with a LTC, adherence was greater in the most deprived areas. Another study found that the effect of behavioural intention (one of the most consistent cognitions positively associated with behaviour [35]) on protective behaviours during the pandemic was moderated by area deprivation; as area deprivation scores increased from low (most deprived) to high (least deprived) the positive impact of behavioural intention on behaviour increased [36].

Why our study did not observe age moderating the relationship between LTC status and adherence is unclear because like gender and area deprivation, age has been associated with adherence [37].

As expected, we found that there was not only a direct relationship between threat appraisal and adherence but people with a LTC or disability increased adherence indirectly through higher threat appraisals in a partial mediation. The PMT framework involves threat appraisal as a critical determinant of behavioural motivation. The greater the perceived threat, the more likely a person will be motivated to protect themselves and ‘protection motivation’ is synonymous with the intention to perform a behaviour [38]. Hence, our findings validate both HBM and PMT which are two psychological theories that position *threat appraisal* as a key cognitive motivational determinant for adopting protective behaviours [16,17].

Finding that adherence is mediated by perceived threat but not psychological distress is compatible with a dual processing approach to dealing with danger. The Common-Sense Self-Regulation Model [39] proposes that when faced with threat, two forms of coping are engaged – one to reduce the emotional response and one to manage the danger per se. Based on our study, it would appear that people with a LTC cope with the threat of COVID-19 by efforts to manage the infection risk and emotional response to that risk (perceived threat).

### 4.1 Implications for public health policy

The findings have several implications for public health policy. First, people with a LTC are likely to be receptive to public health messaging about protective behaviours from infectious diseases. Second, interventions (e.g., public health messaging, vaccination program) that aim to increase adherence in people with a LTC might want to target specific groups in the population that are disproportionally affected by infectious diseases, such as people living in deprived compared to affluent areas. Third, threat appraisal is likely to be an important intervention component because it partially mediates the relationship between LTC status and adherence. Tailored persuasive communication for instance, may include threat messages that do not provoke psychological distress in order to influence adherence in people with LTC(s).

### 4.2 Strengths and limitations

The study benefited from a large sample that was broadly representative of the Scottish UK adults, an investigation of several protective behaviours (face covering, physical distancing, hand hygiene), and *a priori* hypothesises derived from the literature and theories of behaviour. In addition, the random digit dialling made sure the people participating also included people without internet access, with lower literacy, and older population that would be less likely to participate in online surveys. Limitations include the use of self-report measures of behaviour that are open to socially desirable responding.

### 5 Conclusions

Further research about LTC and protective behaviours is critical for informing public health strategies about to infectious diseases such as, COVID-19 and influenza given they are one of the most at-risk groups of the population. Nevertheless, it would appear that people with LTCs tend to act to reduce threat of infection, thus perceptions of threat may be useful intervention targets during future pandemics.

## Ethics approval

All procedures performed in studies involving human participants were in accordance with the ethical standards of the UK National Health Service (NHS) research committee that approved the study and with the 1964 Helsinki declaration and its later amendments or comparable ethical standards. Informed consent was obtained from all individual participants included in the study. Ethical approval for this study was granted by the Life Sciences and Medicine College Ethics Review Board(CERB) at the University of Aberdeen (CERB/2020/5/1942).

## Competing interests

The authors declare that they have no competing interests.

## Funding

Chief Scientist Office(Scotland) (COV/ABN/20/07).

## Authors contributions

GH, CD, DD, MJ designed the study, CD analysed the data, GH drafted the manuscript and CD, DD, MJ commented on and approved the final draft.

## Availability of data and materials

The datasets used and/or analysed during the current study are available from the corresponding author on reasonable request.

## Declaration of competing interest

The authors declare that they have no competing of conflicts of interests.
